# Alteration of serum levels of tumour necrosis factor-a (TNF-a), interleukin-6 (IL-6), C-reactive protein (CRP), gastrin (GAS) and motilin (MTL) after treatment in the Helicobacter pylori-positive gastric ulcer

**DOI:** 10.5937/jomb0-55373

**Published:** 2025-10-28

**Authors:** Yunxiang Chu, Lin Jin, Yan Weng, Xiaochuan Liu

**Affiliations:** 1 Emergency General Hospital, Department of Gastroenterology, Beijing 100028, China

**Keywords:** serum levels of tumour necrosis factor-a (TNF-a), interleukin-6 (IL-6), C-reactive protein (CRP), gastrin (GAS), motilin (MTL), Helicobacter pylori, gastric ulcer, probiotics, quadruple therapy, inflammation, intestinal flora, nivo faktora nekroze tumora-alfa (TNF-a), interleukin-6 (IL-6), C-reaktivni protein (CRP), gastrin (GAS), motilin (MTL), Helicobacter pylori, želudačna ulceracija, probiotici, kvadrupla terapija, inflamacija, crevna flora

## Abstract

**Background:**

This study aimed to explore the clinical efficacy of probiotics plus quadruple therapy on Serum levels of tumour necrosis factor-a (TNF-a), interleukin-6 (IL-6), C-reactive protein (CRP), gastrin (GAS) and motilin (MTL) in treating Helicobacter pylori (Hp)-positive gastric ulcer (GU).

**Methods:**

One hundred and twenty-four patients with Hp-positive GU treated in our hospital from January 2021 to January 2024 were randomly separated into control and observation groups. The former received conventional quadruple therapy, and the latter received tetralogy of viable bifidobacterium tablets combined with conventional quadruple therapy. The clinical efficacy, Hp eradication rate, levels of inflammatory cytokines and gastrointestinal hormone, incidence of adverse reactions, and quality of life were compared in 2 groups.

**Results:**

The total effective rate of the observation group was 96.77%, higher than that of the control group (82.25%). The Hp eradication rate in the observation group was higher than in the control group. After therapy, IL-6, TNF-a and CRP levels declined in 2 groups, and those in the observation group presented lower compared to the control group. After therapy, MLT level was elevated while GAS level was declined in 2 groups, and the improvements of MLT and GAS levels in the observation group were more significant than those in the control group. The incidence of diarrhoea in the observation group was lower than that in the control group, and there was no difference in the incidence of nausea and abdominal distension between 2 groups.

**Conclusions:**

This study investigated the effects of probiotics combined with quadruple therapy on serum levels of tumour necrosis factor-a (TNF-a), interleukin-6 (IL-6), C-reactive protein (CRP), gastrin (GAS), and motilin (MTL) in patients with Helicobacter pylori (Hp)-positive gastric ulcer (GU). The results demonstrated that this combined treatment approach significantly improved Hp eradication rates, reduced inflammatory cytokine levels, and regulated gastrointestinal hormone secretion. Furthermore, patients receiving probiotics with quadruple therapy experienced better maintenance of intestinal flora balance and enhanced overall quality of life than those receiving conventional quadruple therapy alone. These findings suggest that integrating probiotics into standard treatment protocols for Hp-positive GU may offer a safer and more effective therapeutic strategy, addressing inflammation control and gastrointestinal health while improving patient outcomes.

## Abstract (sr)

**Uvod:**

Ova studija imala je za cilj da istraži kliničku efikasnost probiotika u kombinaciji sa kvadruplom terapijom na nivoe faktora nekroze tumora-alfa (TNF-a), interleukina-6 (IL-6), C-reaktivnog proteina (CRP), gastrina (GAS) i motilina (MTL) u serumu u lečenju čira na želucu (GU) i pozitivnog nalaza na Helicobacter pylori (Hp).

**Metode:**

Sto dvadeset i četiri pacijenta sa Hp-pozitivnom želučanom ulceracijom, lečenih u našoj bolnici od januara 2021. do januara 2024. godine, su nasumično podeljeni u kontrolnu i posmatranu grupu. Kontrolna grupa je primala konvencionalnu kvadruplu terapiju, dok je posmatrana grupa primala tablete sa živim bifidobakterijama u kombinaciji sa konvencionalnom kvadruplom terapijom. Klinička efikasnost, stopa eradikacije Hp, nivoi inflamatornih citokina i gastrointestinalnih hormona, učestalost nuspojava i kvalitet života su upoređeni između ove dve grupe.

**Rezultati:**

Ukupna efikasnost u posmatranoj grupi iznosila je 96,77%, što je više u poređenju sa kontrolnom grupom (82,25%). Stopa eradikacije Hp u posmatranoj grupi bila je viša nego u kontrolnoj grupi. Nakon terapije, nivoi IL-6, TNF-a i CRP opali su u obe grupe, a vrednosti u posmatranoj grupi bile su niže u poređenju sa kontrolnom grupom. Nakon terapije, nivo MTL se pove ao, dok je nivo GAS opao u obe grupe, a poboljšanja nivoa MTL i GAS u posmatranoj grupi su bila značajnija nego u kontrolnoj grupi. Učestalost dijareje u posmatranoj grupi bila je niža nego u kontrolnoj grupi, dok nije bilo razlike u učestalosti mučnine i nadimanja između ove dve grupe.&nbsp;

**Zaključak:**

Ova studija je istražila efekte probiotika u kombinaciji sa kvadruplom terapijom na nivoe faktora nekroze tumora-alfa (TNF-a), interleukina-6 (IL-6), C-reaktivnog proteina (CRP), gastrina (GAS) i motilina (MTL) u serumu kod pacijenata sa Hp-pozitivnom želudačnom ulceracijom (GU). Rezultati su pokazali da ova kombinovana terapija značajno poboljšava stope eradikacije Hp, smanjuje nivoe inflamatornih citokina i reguliše sekreciju gastrointestinalnih hormona. Pored toga, pacijenti koji su primali probiotike uz kvadruplu terapiju imali su bolje održavanje ravnoteže crevne flore i poboljšan ukupni kvalitet života u poređenju sa onima koji su primali samo konvencionalnu kvadruplu terapiju. Ova saznanja sugeri{u da integracija probiotika u standardne terapijske protokole za Hp-pozitivnu želudačnu ulceraciju može ponuditi bezbedniju i efikasniju terapijsku strategiju, koja obuhvata kontrolu inflamacije i zdravlje gastrointestinalnog trakta, dok istovremeno poboljšava ishode lečenja pacijenata

## Introduction

Gastric ulcer (GU) is a common digestive tract disease closely related to long-term irregular work, rest, and poor eating habits [1]. Helicobacter pylori (Hp) infection is the most common cause of GU, which can cause pepsin and gastric acid to damage gastric mucosa, induce inflammation, and promote the formation of ulcers [2]. The clinical symptoms of Hp-positive GU are mainly manifested as upper abdominal pain, hematemesis and abdominal distension [3]. The continuous aggravation of the disease may lead to the occurrence of stomach perforation, stomach bleeding, and other related symptoms. It may even lead to stomach cancer, which will bring severe adverse effects on the health and life safety of patients [4]. Because people’s living and eating habits are constantly changing, the number of cases of this disease shows an obvious upward trend [5].Quadruple therapy is the main means of medicine to treat this disease, which can inhibit gastric acid secretion, protect gastric mucosa, and eradicate Hp [6]. However, the large number of antibiotics and irregular use can result in increased drug resistance, a gradual decline in the efficacy of quadruple therapy, and repeated attacks [7]. Studies have shown that probiotic-assisted therapy can effectively improve the eradication rate of Hp, effectively inhibit the growth of Hp, increase intestinal probiotics, promote intestinal peristalsis, and improve gastrointestinal local immunity [8].

The mechanism of probiotics inhibiting Hp can be roughly divided into two categories: non-immune mechanism and immune mechanism [9]. Non-immune mechanisms constitute non-immune barriers against pathogens, such as the acidic environment of the stomach cavity and the mucosal layer barrier of the stomach epithelium [10]. The immune mechanisms of probiotics contain the production of antimicrobial substances (bacteriocins), repression of Hp adhesion to gastric mucosa, induction of mucin production, and stabilisation of the intestinal mucosal barrier [11]. Probiotics can promote the release of antibacterial substances containing lactic acid, acetic acid, short-chain fatty acids, and hydrogen peroxide, thereby reducing the colonisation of stomach bacteria [12]. Probiotics rebalance pro-inflammatory and antiinflammatory cytokines, activate defence mechanisms against pathogens, and induce lymphocyte proliferation. In addition, probiotics induce mucin production, producing metabolites with antimicrobial activity [13].

In this study, we aimed to evaluate the clinical efficacy of combining probiotics with quadruple therapy in treating Hp-positive GU. Specifically, we assessed its impact on Hp eradication rates, inflammatory cytokine levels (TNF-α, IL-6, CRP), gastrointestinal hormone regulation (GAS, MTL), adverse reaction incidence, and overall quality of life. By addressing these parameters, this study provides insights into whether probiotic supplementation can enhance treatment outcomes and offer a more effective and safer therapeutic strategy for Hp-positive GU.

## Materials and methods

### Study design and ethical considerations

This study was conducted as a randomised controlled trial at the Department of Gastroenterology, Emergency General Hospital, Beijing, China, from January 2021 to January 2024. The study protocol received ethical approval from the Ethics Committee of Emergency General Hospital, ensuring that all procedures complied with ethical standards for human research. Informed consent was obtained from all participants before enrollment, and the study adhered to the principles outlined in the Declaration of Helsinki for protecting human subjects in biomedical research.

### Sample size and patient selection

A total of 124 patients diagnosed with *Helicobacter pylori* (Hp)-positive gastric ulcer (GU)were enrolled in this study. The participants were randomly assigned into two groups, each comprising 62 patients. The control group received conventional quadruple therapy, while the observation group received the same quadruple treatment in combination with tetralogy of viable bifidobacterium tablets. The selection of participants was based on strict inclusion and exclusion criteria to ensure the reliability of the study results.

### Implementation method

The inclusion criteria for patient enrollment required individuals 18 years or older with a confirmed diagnosis of Hp-positive GU based on gastroscopy findings and a positive 13C urea breath test. Patients were included if they had no history of severe gastric complications, such as perforation or bleeding, and no known allergies or contraindications to the study medications. Only those with complete and reliable clinical records were also considered for participation.

Patients were excluded from the study if they had severe hepatic or renal dysfunction, a history of gastroduodenal surgery, or had recently used glucocorticoids or NSAIDs. Pregnant or lactating women were also excluded, along with patients diagnosed with recurrent or degenerative ulcers. These criteria ensured a homogeneous study population, minimising external factors that could influence treatment outcomes.

### Treatment protocol

The control group received conventional quadruple therapy. The patient orally took clarithromycin(Zhejiang Jinxin Pharmaceutical Co. Ltd., specification: 0.25 g× 8 tablets/box). The dosage was 0.5 g/time, and the frequency was 2 times/day. The patient was orally given omeprazole (Astra-Zeneca Pharmaceutical Co., LTD., specification: 20 mg × 7 tablets/box). The dosage was 20 mg/time, and the frequency was 2 times/day. The patient took orally amoxicillin (Zhejiang Jinhua Conba Biopharmaceutical Co., LTD., specification: 0.25 g × 24 capsules/box). The dosage was 1 g/time, and the frequency was 2 times/day. The patient took oral colloidal bismuth pectin (Huabei Pharmaceutical Co., LTD., specification: Bi: 50 mg × 24 capcules/box). The dosage was 200 mg/time, and the frequency was 2 times/day.

The observation group received tetralogy of viable bifidobacterium tablets combined with conventional quadruple therapy. The traditional quadruple therapy was the same as the control group, and patients took tetralogy of viable bifidobacterium tablets (Hangzhou Yuanda Bio-pharmaceutical Co., LTD., specification: 0.5 g/piece) orally. The dosage was 1.5–2.0 g/time, and the frequency was 3 times/day.

Both groups received treatment for 2 weeks, next omeprazole for 6 weeks. Then, after 1 month, gastroscopy examinations were performed.

### Observation indicators

(1) Clinical efficacy. Obvious effect: After treatment, the clinical symptoms and signs of the patientsignificantly improved or even disappeared; the ulcer surface formed scars or disappeared, the mucosal oedema disappeared, and the surrounding tissue was not inflamed. Effective: Following treatment, the patient’s clinical symptoms and signs were relieved, the ulcer surface was reduced by more than 50%, the mucosa showed mild oedema, and the surrounding tissue had slight inflammation. Ineffective: Followed by treatment, the patient’s clinical symptoms and signs did not improve, the ulcer surface was reduced by less than 50%, the mucosal oedema was severe, and the surrounding tissue was severely inflamed. Total effective rate = (number of obviously effective cases + number of effective cases)/Total number of cases ×100%.

(2) Hp eradication rate. After treatment, 13C urea breath test was performed on an empty stomach 1 month after drug withdrawal, and the negative result indicated successful eradication of Hp.

(3) Levels of inflammatory cytokines and gastrointestinal hormones. 5 mL fasting venous blood was gathered from patients and centrifuged for 10 min with a radius of 10 cm to obtain upper serum. Serum levels of tumour necrosis factor-α (TNF-α), interleukin-6 (IL-6), C-reactive protein (CRP), gastrin (GAS) and motilin (MTL) were determined by ELISA.

(4) Incidence of adverse reactions containing nausea, abdominal distension, and diarrhoea was recorded in 2 groups.

(5) Quality of life: The 36-item Short Form Health Survey (SF-36) [14] was implemented to evaluate the quality of life from eight dimensions, including physiological function, physical function, bodypain, general health, mental health, emotional function, vitality and social function, with a score of 0-100 for each dimension. A high score indicated a good quality of life.

### Statistical analysis

SPSS 24.0 statistical software was adopted for data analysis. Measurement data were expressed as (x̄±s), and a t-test was adopted for comparison. Count data were expressed as (n, %), and the χ^2^ test was adopted for comparison. P<0.05 meant statistical significance.

## Results

The demographic characteristics of the patients in both groups were analysed to ensure there were no significant differences between them at baseline. The average age of participants in the control group was 46.68±12.38 years, while that of the observation group was 46.72±12.41 years (P=0.985). The distribution of male and female patients was also comparable, with 32 males and 30 females in the control group and 31 males and 31 females in the observation group (P=0.857).

The average duration of disease was 2.17±0.32 years in the control group and 2.21±0.35 years in the observation group (P=0.507), showing no statistical difference. The ulcer locations were similarly distributed, with 35 patients in the control group and 36 in the observation group having ulcers in the gastric antrum, 24 and 22 patients with ulcers in the gastric angle, and 3 and 4 patients with ulcers in the gastric body, respectively (P=0.885). These findings indicate that both groups were well-matched at baseline, ensuring comparability in treatment outcomes.

### Clinical efficacy and Hp eradication

The total effective rate of the observation group was 96.77%, higher than that of the control group (82.25%), with a significant difference (P<0.05) Table 1(Table 2).

**Table 1:** General data of patients in 2 groups.

Items		Control group<br>(n=62)	Observation group<br>(n=62)	χ^2^/t	P
Gender (male/female)		32/30	31/31	0.032	0.857
Age (years)		46.68±12.38	46.72±12.41	0.017	0.985
Course of disease (years)		2.17±0.32	2.21±0.35	0.664	0.507
Ulcer sites	Gastric antrum	35	36	0.243	0.885
	Gastric angle	24	22		
	Gastric body	3	4		

**Table 2:** Clinical efficacy in 2 groups.

Groups	N	Obviously effective	Effective	Ineffective	Total effective rate
Control group	62	20	31	11	51 (82.25%)
Observation group	62	24	36	2	60 (96.77%)
χ^2^					6.960
P					0.008

There were 50 patients with Hp eradication in the control group, the eradication rate was 80.64%, and 60 patients with Hp eradication in the observation group, the eradication rate was 96.77%. The Hp eradication rate in the observation group was higher than the control group (P<0.05, Table 3).

**Table 3:** Hp eradication rate in 2 groups.

Groups	N	Number of Hp eradication	Hp eradication rate
Control group	62	50	80.64%
Observation group	62	60	96.77%
χ^2^		8.052	
P		0.004	

### Levels of inflammatory cytokines and gastrointestinal hormones

**Figure 1:**
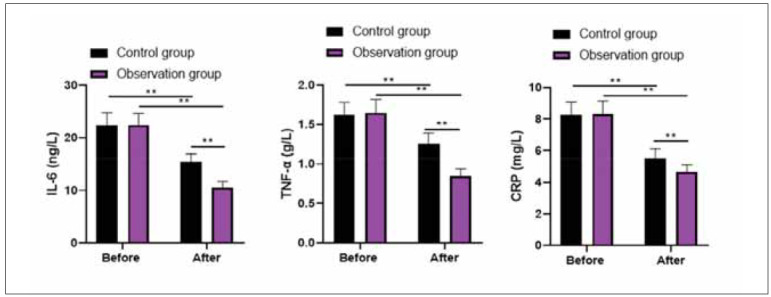
Levels of inflammatory cytokines in 2 groups.<br>**P<0.01

Before therapy, no differences were discovered in IL-6, TNF-α, and CRP levels between 2 groups (P>0.05). After treatment, IL-6, TNF-α, and CRP levels declined in 2 groups, and those in the observation group presented lower than the control group (P<0.05, Figure 1).

**Figure 2:**
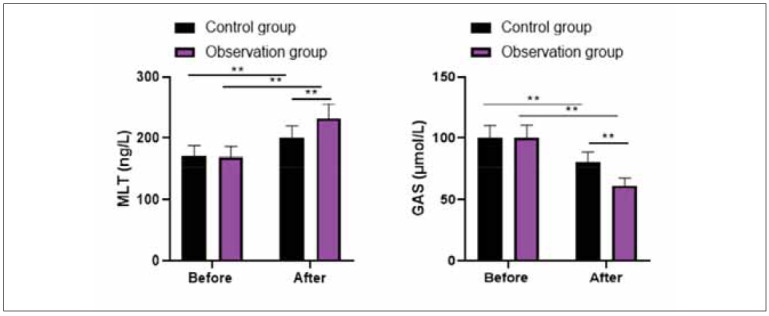
Levels of gastrointestinal hormone in 2 groups.<br>**P<0.01

Before therapy, no differences were discovered in MLT and GAS levels between 2 groups (P>0.05). After treatment, MLT level was elevated while GAS level was declined in 2 groups, and the improvements of MLT and GAS levels in the observation group were more significant than in the control group (P<0.05, Figure 2).

### Incidence of adverse reactions and quality of life

Table 4 displayed that the incidence of diarrhoea in the observation group was lower than that in the control group (P<0.05), and there was no difference in the incidence of nausea and abdominal distension between 2 groups (P>0.05).

**Table 4:** Incidence of adverse reactions in 2 groups.

Groups	N	Nausea	Abdominal distension	Diarrhea
Control group	62	5	3	10
Observation group	62	3	2	2
χ^2^		0.534	0.208	5.905
P		0.464	0.648	0.015

**Figure 3:**
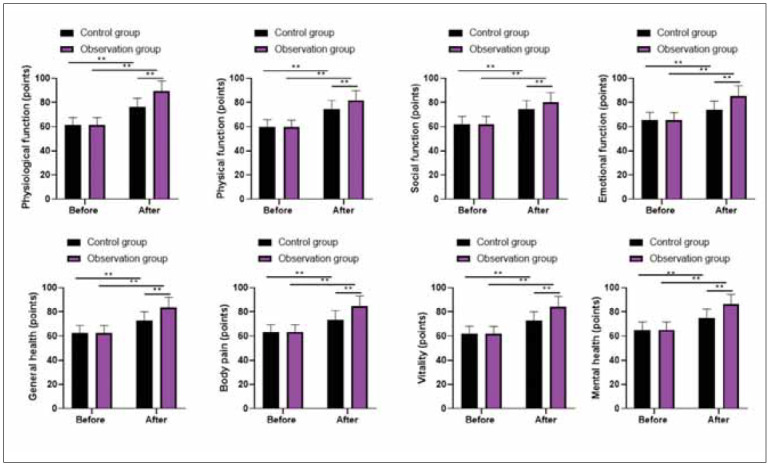
Quality of life in 2 groups.<br>**P<0.01.

Before therapy, no differences were discovered in physiological function, physical function, body pain, general health, mental health, emotional function, vitality and social function scores between 2 groups (P>0.05). Followed by therapy, physiological function, physical function, body pain, general health, mental health, emotional function, vitality and social function scores were elevated in 2 groups, and those in the observation group presented higher as compared with the control group (P<0.05, Figure 3).

## Discussion

GU belongs to a common digestive system disease in the clinic, and about 10% of the population has experienced GU [15]. As Hp infection is an important cause of GU, this kind of GU caused by Hp infection is clinically called »HP-positive GU [16]«. After the onset of GU, patients often have symptoms such as upper abdominal pain, abdominal distension, acid reflux, belching, nausea, etc., which seriously affect the daily lives of patients [17]. If the patient fails to receive timely treatment after the onset of the disease, the ulcer lesions will often continue to expand. Then complications such as digestive tract bleeding and perforation will occur, which has a particular risk of death [18]. Therefore, active treatment of HP-positive GU should be carried out clinically.

At present, quadruple therapy is usually used in clinics to treat HP-positive GU [19]. In the quadruple therapy adopted in this study, omeprazole is a common proton pump inhibitor, which can hinder the secretion of gastric acid, play a specific inhibitory role in the synthesis of adenosine triphosphate, increase the pH value of the stomach, rapidly stabilise enzymesensitive antibiotics, strengthen the bactericidal effect of unstable antibiotics in acidic environment, and provide an ideal environment for the play of antibiotic amoxicillin and clarithromycin [20]. Clarithromycin belongs to macrolide antibiotics, which can inhibit the nuclear protein 50S subunit linkage, hinder protein synthesis and play an antibacterial role [21]. Amoxicillin plays a bactericidal role by inhibiting cell wall synthesis [22]. Colloidal Bismuth Pectin has strong colloidal properties in an acidic environment, which can generate a firm protective film on the surface of gastric mucosa and play a protective role in gastric mucosa [23]. However, some clinical literature has revealed that the impact of quadruple therapy on HP-positive GU is still not satisfactory. Previous reports have suggested that Hp can not only cause direct damage to gastric mucosal epithelium through its virulence protein but also cause an imbalance of gastric and intestinal flora [24].

Tetralogy of viable bifidobacterium tablets is a compound preparation mainly composed ofBifidobacterium infantilum, Lactobacillus acidophilus, Enterococcus faecalis, and Bacillus cereus, which can not only supplement the normal intestinal flora, correct the intestinal microecological imbalance, inhibit the growth and reproduction of pathogenic bacteria, but also help to repair the mucosal barrier function of the gastrointestinal tract [25].

In our study, the results suggested that the total effective rate and the Hp eradication rate in the observation group were higher than those in the control group, suggesting that probiotics plus quadruple therapy effectively treated Hp-positive GU and could promote the Hp eradication rate. The reason may be that the tetralogy of viable bifidobacterium tablets can also promote the release of lactic acid, hydrogen peroxide and other antibacterial substances, kill Hp and inhibit its growth and reproduction [26]. Meanwhile, tetralogy of viable bifidobacterium tablets can also secrete active proteins and other substances to inhibit Hp adhesion and growth, thus inhibiting Hp colonisation and improving the clearance effect [27]. A multicenter randomised, double-blind, placebo-controlled trial investigated the effects of probiotics on side effects during H. pylori eradication therapy. The study demon strated that probiotic supplementation mitigated adverse events associated with eradication therapy and helped neutralise the reduction of gut Bacteroidetes caused by eradication drugs.

Additionally, patients treated with probiotics exhibited smaller fluctuations in gastric microbiota than those receiving a placebo, indicating a stabilising effect on the gastrointestinal environment [13]. Furthermore, a meta-analysis evaluated the efficacy and safety of probiotics in eradicating H. pylori. The analysis concluded that probiotics improved the eradication rate and reduced side effects when added to the treatments designed to eradicate H. pylori [28].

Both Hp infection and GU can cause inflammation and increase the serum pro-inflammatory factor levels [29]. Among them, IL-6 can promote lymphocyte differentiation and antibody production and participate in the damage process of gastric mucosa, and its level is positively correlated with Hp infection [30]. CRP can induce the production of inflammatory cytokines such as TNF-α, and its level is positively correlated with the degree of Hp infection, which can reflect the level of inflammation in the body [31]. In this study, it was revealed that after therapy, IL-6, TNF-α and CRP levels declined in 2 groups, and those in the observation group presented lower as compared with the control group, suggesting that probiotics plus quadruple therapy could repress the inflammation response in the treatment of Hp-positive GU. The reason may be that, on the one hand, tetralogy of viable bifidobacterium tablets can improve the Hp eradication rate as well as reduce the damage of gastric mucosa to reduce the inflammatory response and immune stress caused by bacterial infection and body injury [32]. On the other hand, probiotic bacteria and metabolic components can stimulate the gastrointestinal mucosal immune system, regulate the immune response by regulating dendritic cells and macrophages, improve cellular immune function, and inhibit monocyte production of TNF-α along with other pro-inflammatory factors by affecting T cell differentiation, thus playing a role in immune regulation and anti-inflammatory [33].

Hp infection can damage gastric mucosa, promote the infiltration of gastric antral neutrophils andmonocytes, and make gastric mucosa secrete a large amount of pepsin, resulting in increased serum PG I content, accelerating the erosion of gastric mucosa and affecting the secretion of gastrointestinal hormones [34]. The specific manifestations are increased secretion of GAS and MTL, while excessive GAS and MTL content will promote gastric acid secretion and aggravate ulcers [35]. In our study, the results suggested that after therapy, MLT level was elevated. In contrast, the GAS level was declined in 2 groups. The improvements in MLT and GAS levels in the observation group were more significant compared to the control group, suggesting that probiotics combined with quadruple therapy could improve gastrointestinal hormone levels in treating Hp-positiveGU. The reason may be that tetralogy of viable bifidobacterium tablets can increase the number of beneficial bacteria such as bifidobacterium and Lactobacillus acidophilus in the intestine, directly regulate the imbalance of flora caused by antibiotics, promote the improvement of gastrointestinal function, and improve the secretion of GAS and MTL [36]. At the same time, tetralogy of viable bifidobacterium tablets can also produce beneficial substances such as peptides and amino acids, repair and nourish gastric mucosa and promote the functional recovery of gastric mucosa [37].

In general, the human intestinal flora is relatively stable. Still, applying quadruple therapy will cause changes in the microecological environment and intestinal flora imbalance, leading to repeated disease attacks, so it is essential to observe patients’ intestinal flora in the treatment process [38]. In our study, the incidence of diarrhoea in the observation group was lower than in the control group; decreased diarrhoea incidence suggests that probiotics plus quadruple therapy could effectively maintain the balance of intestinal flora in treating Hp-positive GU. The reason may be that tetralogy of viable bifidobacterium tablets can inhibit Hp infection, reduce Hp urease activity,prevent its invasion and adhesion, supplement the intestinal flora of the body, and maintain the balance of probiotics in the intestine [39].

In addition, our study also indicated that after therapy, the quality of life was elevated in 2 groups.Those in the observation group presented higher than those in the control group, suggesting that probiotics plus quadruple therapy was safe and could promote the quality of life in treating Hp-positive GU, consistent with previous studies [40] [41].

## Conclusions

In conclusion, combined with quadruple therapy, probiotics are an effective and safe treatment forHelicobacter pylori (Hp)-positive gastric ulcer (GU). This combination enhances Hp eradication rates, reduces inflammation, regulates gastrointestinal hormones, maintains intestinal flora balance, and improves patients’ quality of life. Future studies should explore long-term effects and underlying mechanisms to optimise treatment strategies further.

### Author contributions

X.L. contributed to the study’s conception and design. Y.C., L.J. and Y.W. performed data collection, analysis and interpretation. Y.C. wrote the first draft of the manuscript. All authors read and approved the final manuscript.

### Funding

No funding was received to assist with the preparation of this manuscript.

### Data availability

Data for this study can be reasonably obtained from the corresponding authors upon request.

### Ethical approval

The Ethics Committee of Emergency General Hospital approved the protocol.

### Consent to participate and consent to publish

Informed consent was obtained from all the participants involved in the study.

### Conflict of interest statement

All the authors declare that they have no conflict of interest in this work.
